# Persistent pneumothorax after laparoscopic appendectomy in a patient who had undergone radical esophagectomy 5 years before: possible relationship with vulnerability of the hiatus after esophagectomy: a case report

**DOI:** 10.1093/jscr/rjae308

**Published:** 2024-05-18

**Authors:** Seiji Ishikawa, Masakazu Hayashida, Daizo Satoh, Shinji Mine, Izumi Kawagoe

**Affiliations:** Department of Anesthesiology and Pain Medicine, Faculty of Medicine, Juntendo University, 2-1-1, Hongo, Bunkyo-ku, Tokyo 113-8421, Japan; Department of Anesthesiology and Pain Medicine, Faculty of Medicine, Juntendo University, 2-1-1, Hongo, Bunkyo-ku, Tokyo 113-8421, Japan; Department of Anesthesiology and Pain Medicine, Faculty of Medicine, Juntendo University, 2-1-1, Hongo, Bunkyo-ku, Tokyo 113-8421, Japan; Department of Esophageal and Gastroenterological Surgery, Faculty of Medicine, Juntendo University, 2-1-1, Hongo, Bunkyo-ku, Tokyo 113-8421, Japan; Department of Anesthesiology and Pain Medicine, Faculty of Medicine, Juntendo University, 2-1-1, Hongo, Bunkyo-ku, Tokyo 113-8421, Japan

**Keywords:** pneumothorax, laparoscopic appendectomy, esophagectomy, postoperative complication, case report

## Abstract

Postoperative pneumothorax is a well-known but relatively rare complication after laparoscopic surgery. Herein, we report a case of persistent pneumothorax after laparoscopic appendectomy. A 57-year-old male, with a history of minimally invasive esophagectomy and intrathoracic gastric tube reconstruction 5 years before, underwent a laparoscopic appendectomy. A chest X-ray taken on postoperative Day 1 revealed the development of the right pneumothorax, which took more than 3 days to resolve spontaneously. Although the mechanism of postoperative pneumothorax was unclear, it seemed likely that the air that had replaced carbon dioxide in the peritoneal cavity migrated into the thoracic cavity through the esophageal hiatus, which was not covered by the peritoneum or pleura after surgical resection. The present case, together with our previous similar case, suggests that a history of esophageal cancer surgery *per se* increases the risk of pneumothorax after laparoscopic surgery, probably regardless of when this was performed.

## Introduction

Pneumothorax after laparoscopic surgery is a well-known but relatively rare complication, with a reported frequency of 0.01%–1.9% [1–3]. We previously reported a case of pneumothorax that developed after laparoscopic surgery and persisted for more than a week in a patient who had undergone esophagectomy 4 months earlier, and concluded that esophagectomy, at least performed recently, may increase the risk of pneumothorax after laparoscopic surgery [4]. After the presentation of the previous case [4], we experienced a similar case.

Herein, we report another case of persistent pneumothorax after laparoscopic surgery in a patient who had undergone esophagectomy, but much earlier than 4 months. This case suggests that a history of esophagectomy *per se* can increase the risk of pneumothorax after laparoscopic surgery, regardless of when esophagectomy was performed.

## Case report

**Figure 1:**
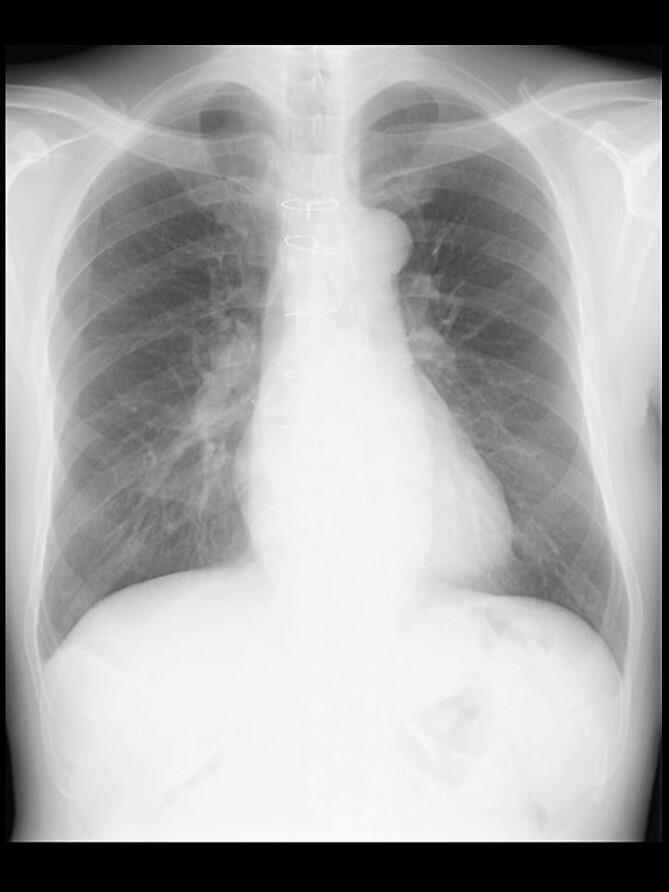
A chest radiograph in the upright position before surgery.

A 57-year-old male, 178 cm and 69 kg, was scheduled for a laparoscopic appendectomy for chronic appendicitis. The patient had undergone mitral valve repair for infective endocarditis via a median sternotomy 16 years before and minimally invasive esophagectomy (MIE) with intrathoracic anastomosis using a gastric tube for esophageal cancer 5 years before. Preoperative tests, including a chest radiograph (Fig. 1), were unremarkable. The computed tomography (CT) revealed no emphysematous lung cyst. Anesthesia was induced with intravenous remifentanil (0.3 μg/kg/min) and propofol (100 mg). Intravenous rocuronium (50 mg) was administered to facilitate tracheal intubation. Anesthesia was maintained with inhaled sevoflurane (1.5%) and intravenous remifentanil (0.1–0.3 μg/kg/min). The lungs were ventilated with a volume-guarantee pressure-controlled mode employing an inspired oxygen concentration of 35%. Percutaneous arterial oxygen saturation (SpO_2_) was maintained at 96%–100%. In addition to local anesthetic wound infiltration, intravenous fentanyl (300 μg) and acetaminophen (1000 mg) were administered for immediate postoperative analgesia. Operation and anesthesia times were 121 and 156 min, respectively. The infusion volume was 1350 ml. Emergences from anesthesia and extubation were uneventful. SpO_2_ in the postanesthesia care unit was maintained above 95% without supplemental oxygen. The patient did not complain of chest pain or dyspnea.

**Figure 2:**
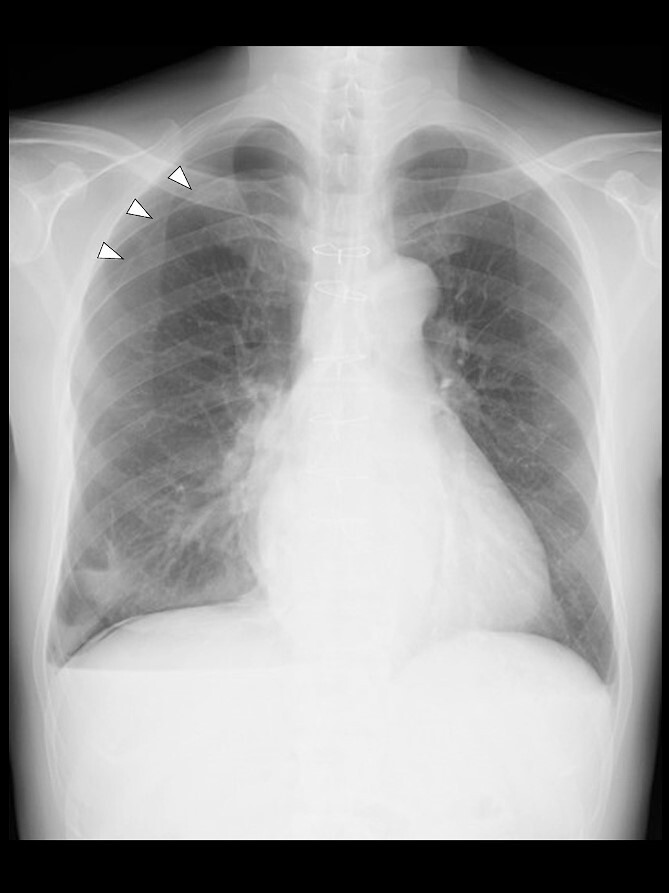
A chest radiograph in the upright position on POD 1 showing a right pneumothorax (arrowheads).

**Figure 3:**
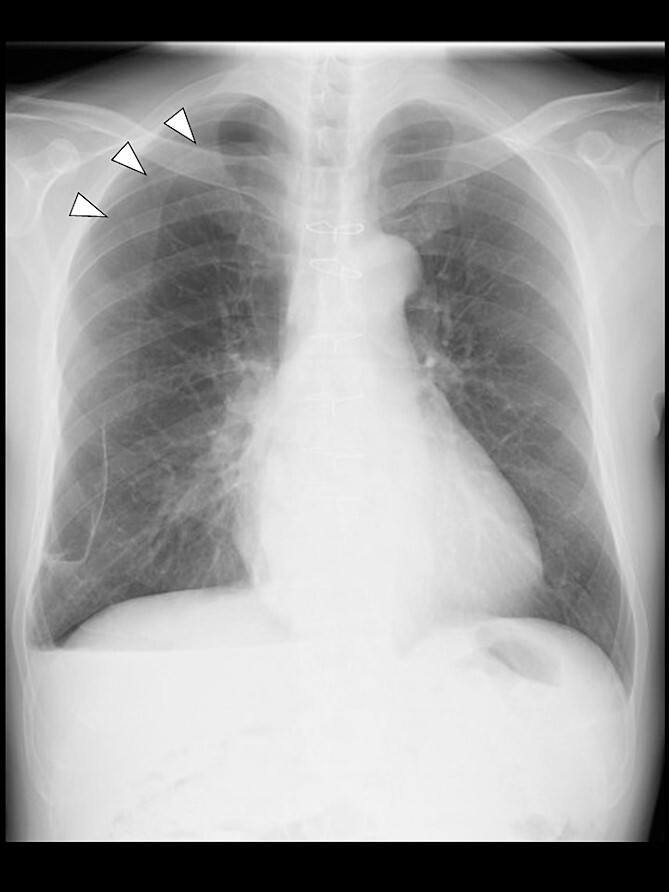
A chest radiograph in the upright position on POD 3 showing a residual right pneumothorax (arrowheads).

Even though the patient did not receive supplemental oxygen in the ward, SpO_2_ remained at 98%. Chest radiographs were not taken on the day of surgery. However, a chest radiograph taken in the upright position on postoperative Day (POD) 1 revealed a pneumothorax on the right side (Fig. 2), although he had no respiratory symptoms. Radiographs on PODs 2 and 3 revealed the pneumothorax remaining unchanged (Fig. 3). The patient was discharged from the hospital on POD 3 without any respiratory symptoms. A chest radiograph taken on POD 19 showed no remaining pneumothorax.

## Discussion

Most pneumothorax after laparoscopic surgery occurs during or immediately after surgery and disappears quickly without the need for specific treatment [5, 6]. Until our previous case report was published [4], only one report and no report had described pneumothorax becoming evident the day after laparoscopic surgery [7] and pneumothorax persisting for days after laparoscopic surgery, respectively.

Known mechanisms of pneumothorax after laparoscopic surgery include iatrogenic damage to the diaphragm [8], rupture of a bulla, and migration of carbon dioxide into the thoracic cavity through subcutaneous emphysema [9]. However, none of these mechanisms seemed applicable to our case, as no bulla was detected preoperatively, the diaphragm was not surgically manipulated, or no subcutaneous emphysema was observed perioperatively.

Similarly to our previous case [4], a pneumothorax was evident after laparoscopic surgery on POD 1 and persisted for days. The long-lasting pneumothorax suggested that the gas trapped in the thoracic cavity was air rather than carbon dioxide [4]. Although the precise mechanism of postoperative pneumothorax in this case was unclear, it seemed likely that the intraabdominal gas migrated into the thoracic cavity through the esophageal hiatus not covered by the peritoneum nor pleura, as esophagectomy and conduit reconstruction involve a large incision in the pleura adjacent to the esophagus and a partial incision in the peritoneum covering the diaphragm. These procedures should leave a transdiaphragmatic communication between abdominal and thoracic cavities, as could be suggested by developments of postesophagectomy diaphragmatic hernias (PEDHs) as an extreme manifestation of this communication, with a reported frequency of 0.7%–19.6% [10].

It is expected that a transdiaphragmatic communication via a hiatal or peri-conduit route would gradually close owing to tissue adhesion, since PEDHs most frequently develop within 2 years after esophagectomy, and thereafter, their occurrences decrease with the elapse of time [11, 12]. However, PEDHs can occur several years after esophagectomy in a proportion of patients [11, 12], and even more than 10 years after esophagectomy [13]. Further, an increase in intraabdominal pressure can cause a sudden PEDH by reopening a previously closed transdiaphragmatic communication [14]. Because a major transdiaphragmatic communication allowing migration of abdominal organs can persist or reopen several to more than 10 years after esophagectomy [11–13], a minor transdiaphragmatic communication allowing migration of the intraabdominal gas would persist much longer and much more frequently and/or would be much more easily reopened by increased intra-abdominal pressure from pneumoperitoneum. Indeed, the present case suggests that even 5 years after esophagectomy, gas migration can occur following laparoscopic surgery. Further, it should be noted that our previous [4] and present patients had undergone MIE, given that the incidence of PEDHs is higher after MIE than after open esophagectomy (6.5% vs. 2.4%) [15].

The above-mentioned data regarding PEDHs and our previous and present cases of persistent pneumothorax after laparoscopic surgery in postesophagectomy patients suggest that a history of esophagectomy, especially that of MIE can increase the risk of pneumothorax after laparoscopic surgery, regardless of the time elapsed since esophagectomy. Clinicians should be aware of the possible development of persistent pneumothorax after laparoscopic surgery in postesophagectomy patients.

In conclusion, the present case suggests that a history of esophagectomy, especially that of MIE can increase the risk of pneumothorax after laparoscopic surgery regardless of the time after esophagectomy, although further studies are required to elucidate whether previous esophagectomy actually increases the risk of pneumothorax after laparoscopic surgery.

## Data Availability

Data sharing is not applicable to this article as no datasets were generated or analyzed during the current study.
